# Draft Genome Sequence of a New Zealand Rickettsia-Like Organism Isolated from Farmed Chinook Salmon

**DOI:** 10.1128/genomeA.00503-16

**Published:** 2016-06-30

**Authors:** Edna Gias, Jenny Draper, Cara L. Brosnahan, Della Orr, Andrew McFadden, Brian Jones

**Affiliations:** Investigation and Diagnostic Centres and Response, Wallaceville, Ministry for Primary Industries, Upper Hutt, New Zealand

## Abstract

We report here the draft genome sequence of a rickettsia-like organism, isolated from a New Zealand Chinook salmon farm experiencing high mortality. The genome is approximately 3 Mb in size, has a G+C content of approximately 39.2%, and is predicted to contain 2,870 coding sequences.

## GENOME ANNOUNCEMENT

Rickettsia-like organisms (RLOs) are Gram-negative bacteria increasingly recognized as important fish pathogens in the salmonid aquaculture industry. *Piscirickettsia salmonis* is the first described RLO affecting fish and causes a systemic infection known as piscirickettsiosis (1). *P. salmonis* was initially isolated in farmed coho salmon, *Oncorhynchus kisutch*, during a high mortality epizootic in Chile in 1989 that resulted in huge economic losses (2, 3). Occurrences of rickettsial septicemia have been reported in farmed salmonids in many countries (4–8) and in various nonsalmonid species (9–12).

A high mortality event among Chinook salmon, *Oncorhynchus tshawytscha*, farmed in the Marlborough Sounds, New Zealand, was reported to the Ministry for Primary Industries in April 2015. During this event an RLO was isolated from the affected fish, of which identification was confirmed by histopathology, PCR, and nucleotide sequencing of the internal transcribed spacer and 16S genes (C. L. Brosnahan et al., unpublished data). We report here the draft genome sequence of NZ-RLO, the first RLO reported from farmed salmonids in New Zealand.

NZ-RLO was isolated from the kidney tissue of an affected Chinook salmon in an epithelioma papulosum cyprinid (EPC) cell line at 15°C, followed by multiple passages in the same cell line at 22°C. When the EPC showed 100% cythopathic effect, the culture was inoculated onto cysteine heart agar supplemented with blood and incubated at 22°C to isolate bacterial colonies (13). Genomic DNA extracted from the bacterial colonies was subjected to shotgun pyrosequencing on a Roche GS Junior 454, according to the manufacturer’s protocol for the XL+ chemistry (Roche). A total of 152,670 reads with an average length of 611 bp were assembled by the GS *de novo* assembler version 2.7. Contigs less than 200 bp in length were discarded. The draft genome has a total length of 3,052,667 bp (463 contigs; *N*_50_, 19,218; longest contig, 58,138 bp) and a GC content of 39.2%. The average depth of coverage was approximately 30×.

Annotation of the contigs was performed with Prokka version 1.11 (14) using a default similarity *e*-value cutoff of 1 × 10^−6^. There were 1,755 and 1,115 coding sequences predicted to encode known proteins and hypothetical proteins, respectively. Features identified in published *P. salmonis* genome sequences (15–17) that were present in NZ-RLO include genes encoding proteins for type IV pili, components of toxin-antitoxin modules (PasT/PasI, PhdYefM/PIN domain-containing toxins, MqsR/MqsA, and MazF/MazE), multiple transposable elements, and proteins for type I and IV secretion systems. Genes encoding a poly-β-hydroxybutyrate granule biosynthesis pathway were also identified. The presence of flagellar components and chemotaxis proteins suggests that NZ-RLO is a motile bacterium. Bacteriophage genes were also present, which is consistent with the prior observation of phage particles associated with *P. salmonis* (18). This genome sequence will allow comprehensive comparison and phylogenetic analysis of NZ-RLO, *P. salmonis*, and other RLOs.

### Nucleotide sequence accession numbers.

This whole-genome shotgun project has been deposited at DDBJ/ENA/GenBank under the accession number LVCQ00000000. The version described in this paper is the first version, LVCQ01000000.
